# Microfluidics for understanding model organisms

**DOI:** 10.1038/s41467-022-30814-6

**Published:** 2022-06-09

**Authors:** Nolan Frey, Utku M. Sönmez, Jonathan Minden, Philip LeDuc

**Affiliations:** 1 Department of Biological Sciences, Carnegie Mellon University, Pittsburgh, PA USA; 2 Department of Mechanical Engineering, Carnegie Mellon University, Pittsburgh, PA USA; 3 Department of Biomedical Engineering, Carnegie Mellon University, Pittsburgh, PA USA; 4 Department of Computation Biology, Carnegie Mellon University, Pittsburgh, PA USA; 5 Department of Electrical and Computer Engineering, Carnegie Mellon University, Pittsburgh, PA USA

**Keywords:** Biotechnology, Biological techniques, Microfluidics, Experimental organisms

## Abstract

New microfluidic systems for whole organism analysis and experimentation are catalyzing biological breakthroughs across many fields, from human health to fundamental biology principles. This perspective discusses recent microfluidic tools to study intact model organisms to demonstrate the tremendous potential for these integrated approaches now and into the future. We describe these microsystems' technical features and highlight the unique advantages for precise manipulation in areas including immobilization, automated alignment, sorting, sensory, mechanical and chemical stimulation, and genetic and thermal perturbation. Our aim is to familiarize technologically focused researchers with microfluidics applications in biology research, while providing biologists an entrée to advanced microengineering techniques for model organisms.

## Abstract (web summary)

Building small-scale tools for biology research eliminates the need for time-consuming methods and enables novel experimental paradigms. Here, the authors discuss microfluidics' potential for manipulating or stimulating model organisms and identify barriers to making these tools accessible.

## Introduction

Understanding how biological organisms function as a system at subcellular, cellular, multicellular, and macroscopic levels is one of modern biology's grand challenges. Genetic, epigenetic, and environmental cues collectively regulate biological processes in a spatiotemporally dependent manner. These cues synchronously control and execute complex biological processes required for development, motility, and perception in an astonishingly robust way across many different species^1^. Understanding the fundamental mechanisms of biological systems equips us to better understand human pathologies and engineer biotechnologies to address societal challenges^2^. To gain insights into the mechanisms underpinning biological processes, researchers utilize model organisms to investigate biological phenomena in endogenous contexts^3^. For over a century, biologists have employed organisms such as *Caenorhabditis elegans* (nematode)*, Drosophila melanogaster* (fruit fly)*, Danio rerio* (zebrafish)*, Xenopus* (clawed frog), and *Arabidopsis thaliana* (rockcress) as models^4^. Insights from these organisms can be extended to other species due to genetic homology and have catalyzed breakthroughs in human health and fundamental biology principles^5,6^. Using these model organisms, researchers have access to longstanding communities with well-annotated -omics databases, tractable genetic systems, mutant libraries, extensive bodies of research, and readily available tools and techniques. Despite these advantages, working with these model organisms using conventional methods can be time-consuming, limits experimental design, and requires expertise that is not easily acquired^7,8^. Consequently, protocols for handling, imaging, or applying various experimental stimuli introduce considerable variability and are typically low-throughput, among other challenges.

**Fig. 1:**
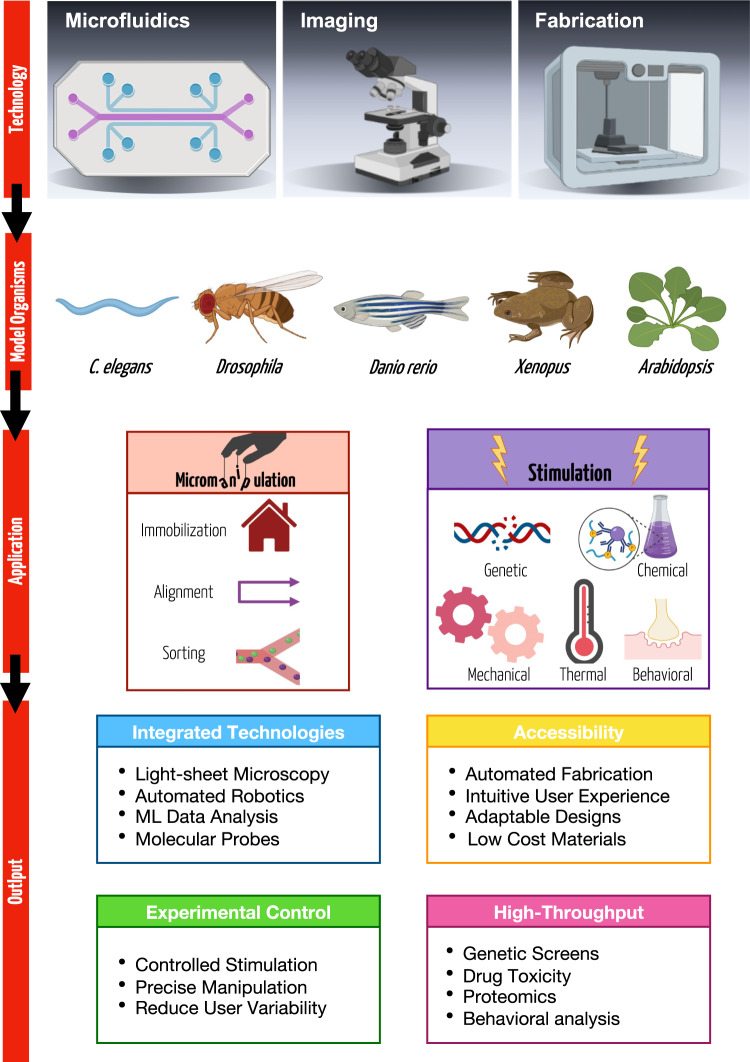
Future transdisciplinary directions for biological model organism research enabled by novel microfluidic approaches. Novel microfabrication techniques combined with creativity enable the production of different microfluidic tools that, in turn, facilitate the development of novel experimental paradigms to be used with small multicellular model organisms. Microfluidics empower researchers with tremendous control over sample stimulation while also enabling precise manipulation of samples, and being compatible with high-resolution live imaging. The potential of these microtechnologies is accelerating as they are integrated with additional technologies, become more accessible, and are designed to be multifunctional. Figure created with BioRender.com.

To address these limitations, researchers employing these model organisms have collaborated with engineers to develop novel microfluidics-based solutions (Fig. 1)^9^. The number of these interdisciplinary studies has skyrocketed in the past two decades. Recent advancements in microfluidic systems demonstrate that they are an excellent resource for designing novel biological assays with precisely controlled experimental parameters, a capacity for high-throughput studies, and the ability to be combined with other technologies. At such a small scale, investigators often have better control over spatiotemporal variables, precise flow regimes, and easier integration with automation tools^10^. Additional benefits achieved at the micrometer scale include a reduced sample volume, predictable mass or energy transfer, portability, and the capacity to run multiple assays in parallel toward high-throughput capabilities, which are critical in many types of experiments from -omics to, behavioral studies, genetic screens, or computational approaches requiring massive data sets. Furthermore, the use of flexible, oxygen permeable, and translucent polymers, such as polydimethylsiloxane (PDMS), which can be designed with complex geometries, enables experimental paradigms previously not imaginable. Precise control of flow through such systems and the ability to incorporate actuators gives experimentalists unparalleled control of a given stimulus. Innovations in materials science and fabrication methods continue to broaden the possibilities for such microfluidic designs while simultaneously making these technologies more accessible to extant biological research communities^11^. Finally, systems initially designed for a particular model organism can be readily scaled or modified for use with other model organisms, inspiring similar experimental paradigms across species^12^.

The work reviewed and discussed for future directions herein applies microfluidic systems to enable higher throughput, improved repeatability, precise stimulation, and automated sorting and alignment. This technology has great potential to enhance standardization across labs, pave the way for developing new experimental paradigms, and automate experimental setups by incorporating other advanced techniques such as light-sheet microscopy, robotics, and machine learning approaches with microfluidics. We present these discussions organized into four categories: (a) precise manipulation, (b) controlled stimulation, (c) design considerations, and (d) future perspectives and outlook. This perspective aims to familiarize engineers with biological organisms while introducing the most recent microengineering approaches to biologists to promote research interactions. This work will lead to furthering the development of novel microfluidic systems that will help solve challenging biological problems through technological innovation.

## Precise manipulation

Microfluidics systems for precise manipulation of model organisms reduce or even eliminate the need for manual handling, consistently positioning samples with precision, and enabling higher throughput analysis and treatment. Commonplace biological approaches, such as microscopy, microinjection, or selecting individuals with specific characteristics from a population, require that samples are physically sorted, aligned, and or immobilized. Manually performing these tasks introduces user variability, requires expertise, and is often time-consuming, resulting in lower sample numbers and higher experimental noise. Below, we analyze microfluidics systems used to immobilize, align, and sort samples at high throughputs with minimum user intervention.

## Immobilization

**Fig. 2:**
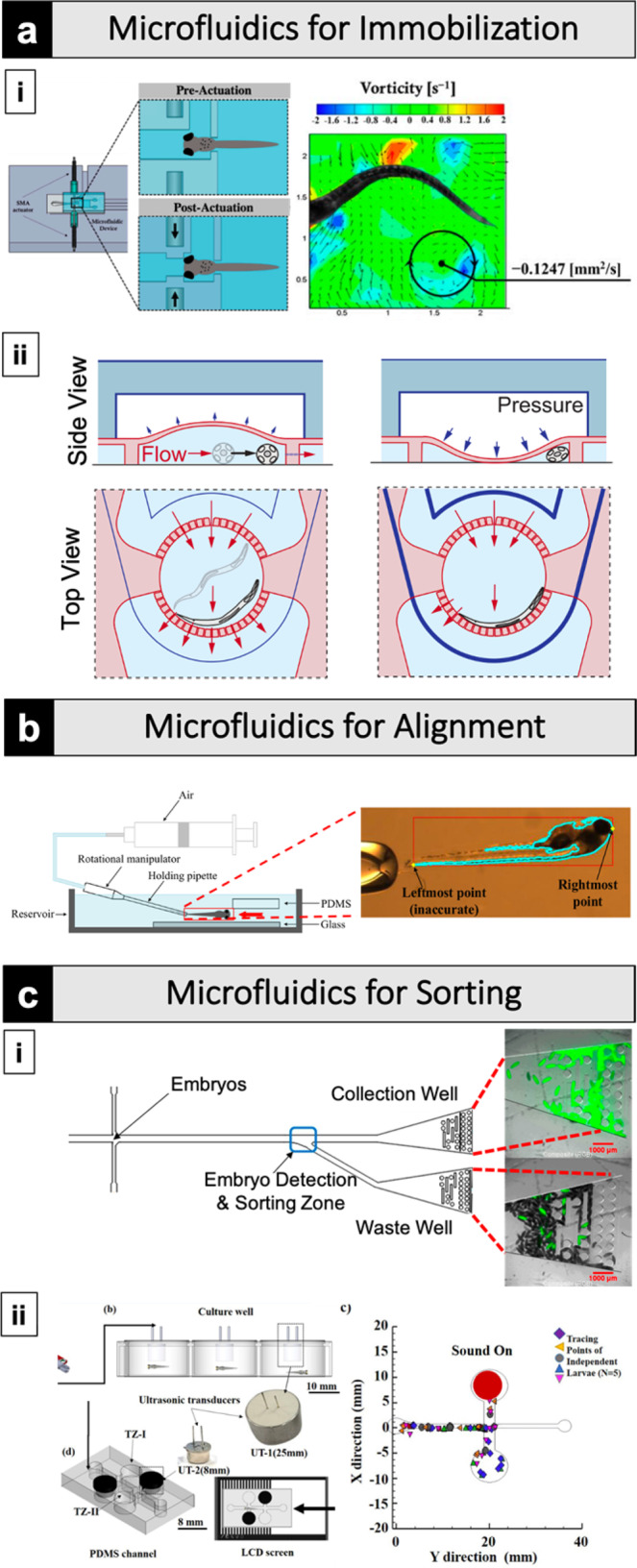
Microfluidics for precise manipulation of model organisms reduce or even eliminate the need for manual handling, consistently positioning samples with precision, and enabling higher throughput analysis and treatment. **a-i** A microfluidic device with a shape memory alloy actuator immobilized zebrafish and examined the hydrodynamic flow resultant from tail beating^21^. **a**-**ii** A worm chamber for visualization and periodic immobilization of *C. elegans*^24^. **b** A microfluidic device coupled with a live detection system designed to gently immobilize, orient, and inject zebrafish larvae^26^. **c**-**i** A microfluidic system to sort fruit fly samples at high throughput and enrichment ratio^29^. **c**-**ii** A non-invasive zebrafish larvae sorting system based on microfluidics that utilized light and acoustics to corral individual samples^34^. All panels are cropped and adapted versions of the originals. Panel **c**-**ii** was reproduced from Mani and Chen^34^, with the permission of AIP Publishing.

Immobilization of individual, whole, live organisms is critical for numerous experiments. Yet, conventional immobilization techniques such as using anesthetics or adhesives are often harmful to the organism, are time-consuming, have low throughput, require experience, and introduce unintended variables. Numerous microfluidic systems have been developed that achieve sample immobilization via physical confinement, controlled delivery of anesthetics, or both to address these longstanding experimental limitations^13–20^. An example of this can be seen in recent work from Subendran et al., wherein they developed a microfluidic device with an integrated shape memory alloy actuator to immobilize zebrafish in an observation region to examine the hydrodynamic flow resultant from tail beating (Fig. 2a-i)^21^. With a similar goal for immobilization, separate work by Chaudhury et al. utilized both cooler temperatures and physical confinement to anaesthetize and subsequently image *Drosophila* larvae^22^. Their design accommodated larvae in a 5.0 mm by 1.6 mm sample microchamber that sits below a second microchamber filled with coolant with the two chambers separated by a thin PDMS layer. Immobilization of larvae was achieved via pressurizing the coolant chamber causing deflection of the separating PDMS layer and, in doing so, slightly compressed and cooled the larvae for imaging through the bottom glass surface. Immobilization by cooling is well suited for live imaging as the reduced temperatures minimize muscle contractions and internal fluid flow which can complicate high-resolution imaging^19^. Both examples achieve immobilization of highly motile samples for imaging without causing physiological damage to the sample. These approaches highlight microfluidics devices' potential to simplify sample immobilization while also enabling higher throughputs and reducing harm to the organism.

Similar approaches can be utilized for long-term immobilization and repeated high-resolution imaging of samples. For example, Martinez et al. were able to investigate the auxin-inducible degron system in *C. elegans* using a microfluidic setup that immobilizes samples via compression for subsequent imaging using a 40× objective on a spinning disc confocal microscope (Fig. 2a-ii)^23,24^. Notably, this setup enabled the researchers to track and visualize dozens of individual worms over multiple days, improving both throughput and temporal analysis. The utility of microfluidic systems for long-term immobilization is further highlighted in the work by Sun et al. utilizing *Arabidopsis*. The influence of high salinity stress on different parts of the root structure was dynamically tracked with high temporal and spatial resolution using a microfluidic device with crossed microchannels^25^. These examples highlight microfluidic systems’ ability to enable longer-term studies. Such methods can produce massive data sets over time from which many biological insights can be gleaned.

## Automated alignment

Proper sample alignment of model organisms is essential for imaging, injection, and external stimulations. Using manual alignment is very challenging in numerous areas and automated alignment will allow for high-throughput approaches to standardize the environment and stimulation, which will be tremendously beneficial for the future. Conventionally, samples are manually aligned with tweezers or paintbrushes brushes which is time-consuming and delicate work that can introduce variability in experiments. Here, we present a recent example of an elegant microfluidic system that automates previously cumbersome alignment processes. Zhang et al. developed a microinjection system for zebrafish larvae organ injection^26^. The system precisely manipulated zebrafish embryos with a V-shaped electrothermal micro-actuator that was integrated into a robotic micromanipulation system. The system gently positioned and oriented embryos with minimal deformation to prevent physiological damage (Fig. 2b). Already capable of injecting tens of individuals at a time, the design is being further optimized to improve efficiency and enable higher throughput. Microinjections are routine across model organism research; microfluidic systems such as the one described here are a promising complement to traditional bench work as they can automate injections to save time and reduce variability within and between labs^13,27^. Some degree of mechanical stress and physiological damage are realities of any injection. Microfluidic systems designed to minimize these harmful effects have the potential to minimize the damage as well as significantly reduce injection variability in addition to the apparent time-savings and throughput advantages they offer. In general, systems designed with versatility and low barriers to entry will accelerate the pace of model organism research and enable readily adaptable experimental paradigms across species for comparative biology.

## Sorting

Sorting model organisms by developmental age or other phenotype is a prerequisite step for many experiments and is conventionally accomplished manually when selecting phenotypes of interest. For example, manually sorting samples by developmental stage is a common prerequisite for many experiments. Microfluidic systems have been developed to accomplish, and in some cases fully automate, sorting samples on a scale unachievable with conventionally implemented manual sorting techniques^19,28^. Utharala et al. created an improved microfluidic platform with integrated valves mounted to a fluorescence microscope to sort *Drosophila* embryos expressing a fluorescent marker with 99% sorting accuracy and 92% sorting efficiency at 0.13 Hz sorting frequency (~470 embryos/h) (Fig. 2c-i)^29^. These types of microfluidics-enabled, large-scale, fluorescent-based mutant screening or phenotype-selection experiments are not feasible with manual sorting. The platform was designed with relatively low-cost components and can collect and separate samples with different characteristics such as fluorescence intensity or color. Droplet-based microfluidics technologies, which utilize two immiscible phases to handle small-volume droplets, are increasingly being applied to model oganisms^30–32^. Aubry et al. utilize Pluronic hydrogel as one of the phases in a droplet-based system. The reversible gelling property of Pluronic enabled them to temporarily immobilize droplet-isolated *C. elegans* larvae for imaging followed by subsequent sorting^33^. The design and throughput of similar sorting devices will permit researchers in model organism communities to carry out investigations previously only possible with cell cultures and fluorescence-activated cell sorting (FACS).

Motile organisms make the task of sorting significantly more challenging. Mani and Chen's recent design exemplifies how zebrafish larvae' motility is leveraged to accomplish clever sorting (Fig. 2c-ii). Their design took advantage of zebrafish larvae's natural aversion to light and acoustics to non-invasively corral the larvae into specific chambers^34–37^. By incorporating a motile animal's natural behavior into their designs, researchers can develop sophisticated automated systems for sorting and analysis, as in the example above. We expect such approaches to accelerate a diversity of areas, including drug screening, mutagenesis analysis, and other similar large-scale studies in model organisms that could benefit from such automation.

## Controlled stimulation

Controlled stimulation can be a powerful approach to investigate biological systems. Manually applied stimulation is inherently limited and can result in experimental variability, making such experiments challenging. Microfluidic systems can deliver stimulations with high precision and at throughputs not previously attainable while also making once laborious experiments less demanding. Modern microfabrication approaches allow for complex geometries utilizing materials with properties tuned to the experimental system. Moreover, the ability to control flow and gradients at small scales provides exquisite control over experimental parameters. Below, we discuss recent experimental paradigms enabled by microfluidic technologies related to the following categories: sensory and behavioral analysis, mechanical stimulation, genetic perturbation, chemical perturbation, and thermal perturbation.

## Sensory stimulation and behavioral analysis

High-throughput behavioral assays are valuable to study the neurological basis of behavior and perception^38,39^. The throughputs enabled by microfluidic systems and the ability to precisely control sensory stimuli coupled with automated data acquisition make microfluidic systems powerful tools for neurological studies in model organisms.

**Fig. 3:**
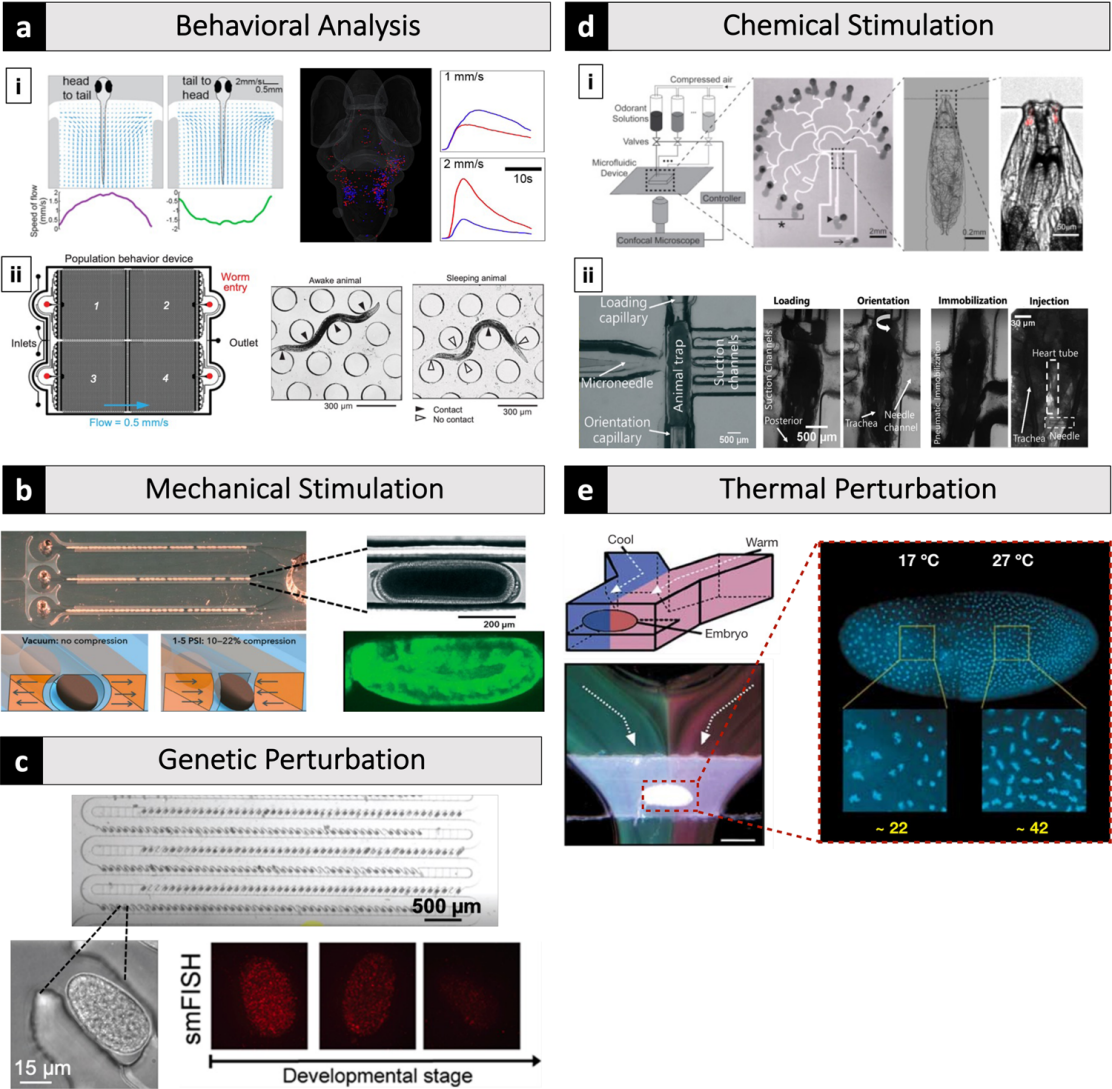
Microfluidic systems can deliver stimulations with high precision and at throughputs not previously attainable while making once laborious experiments less demanding. **a**-**i** A 3D-printed microfluidic device compatible with a custom-built light-sheet microscope to stimulate zebrafish with precise flow vectors for brain-wide calcium imaging^40^. **a**-**ii** A microfluidic device with an array of microposts for analysis of sleep behavior of *C. elegans*^46^. **b** A microfluidic system that automatically aligned *Drosophila* embryos and precisely compressed them using pneumatically actuated deformable sidewalls with simultaneous live imaging^57^. **c** A microfluidic system capable of trapping hundreds of *C. elegans* embryos quickly and enabling efficient reagent exchange^59^. **d**-**i** A microfluidic system visualized the response of olfactory receptor neurons (ORNs) of *Drosophila* larva in response to controlled odorant exposure^69^. **d**-**ii** A microfluidic device with integrated glass capillaries and a microneedle for chemical injection of *Drosophila* larvae^70^. **e** A microfluidic device exposed *Drosophila* embryo to a thermal gradient along the anterior-posterior axis using two laminar flow streams with different temperatures^76^. All panels are cropped and adapted versions of the originals. Panel **c** is adapted with permission from Charles et al.^59^, copyright 2020 American Chemical Society.

A recent design by Vanwalleghem et al. highlights the potential for microfluidics coupled with other emerging technologies, to enable entirely new experimental paradigms for neurological investigation^40^. In this, perceiving water flow is critical for an aquatic animal, and understanding the associated neurons allows researchers to better map this model organism's neurological network. Many experiments investigating sensory systems for flow to date detected neuronal responses with surgically implanted electrodes while stimulating the system with water vibrations or by ablating components of sensory systems and analyzing subsequent behavior^41–43^. In contrast, Vanwalleghem et al. utilized a microfluidic system to stimulate the fish with the controlled flow while simultaneously recording neuronal firing in a non-invasive manner to investigate which neurons in zebrafish are responsible for detecting or processing specific features of fluid flow information. To accomplish this, the team designed a 3D-printed microfluidic device compatible with a custom-built light-sheet microscope to stimulate immobilized zebrafish with precise flow vectors (Fig. 3a-i). High-volume, light-sheet microscopy enabled simultaneous analysis of neuronal spiking events coupled to flow stimulation, as detected via a genetically encoded calcium reporter. Moreover, the team utilized a machine learning approach to decode and categorize the neuronal responses elicited by specific stimuli to analyze the resultant massive dataset. This approach characterized the response of individual neurons to various features of water flow and empowered the group to model the underlying neuronal network. Such a large-scale analysis, made possible by microfluidics coupled with new technologies in molecular biology, machine learning, and microscopy, enabled the team to better observe the structure and function of the brain network in a novel manner.

Microfluidics are also powerful tools to analyze behaviors in large populations of model organisms and for volumetric imaging of organims^44,45^. For example, Lawler et al. utilized a microfluidic device with an array of 200 μm microposts around that motile worms can navigate. Simultaneous imaging and tracking of ~100 worms allowed the researchers to analyze sleep behaviors defined by prolonged contact with the microposts (Fig. 3a-ii)^46,47^. The microfluidic device also enabled the researchers to investigate the influence of environmental factors such as fluid flow, oxygen levels, temperature, or specific odorants by readily altering the fluid dynamics and characteristics within their system as others have demonstrated^48^. Highlighting the scalability of microfluidics, the Lawler group fabricated a smaller system to track and analyze individual worms. This smaller setup was utilized, in part, to visualize neuronal activity using a genetically encoded calcium reporter to characterize the behavior of the neuron network during different sleep states and developmental stages with single neuron-resolution. This work highlights the incredible versatility of microfluidics systems in the area of sensory stimulation and behavioral analysis while also providing an example of such technologies' potential when coupled with other modern technologies.

## Mechanical stimulation

Physical forces play a central role in development and physiology across almost all organisms^49–51^. Various techniques including indentation with pipettes, compression with a piezoelectric controlled glass slide, and magnetic tweezers have been utilized to investigate the biological response to mechanical stress^52–54^. Microfluidic systems can generate different modes of spatiotemporally controlled mechanical stimulations that can be used for novel quantitative assays to examine the effects of mechanical stress in a more controlled and high-throughput manner as compared to these conventional approaches^55,56^. For instance, we developed a microfluidic system that automatically aligned hundreds of *Drosophila* embryos into an end-to-end alignment along their anteroposterior axis without using any external flow equipment^57^. In this approach, embryos were aligned into channels and loaded through gravity by tilting the microdevice. Hundreds of *Drosophila* embryos were loaded into microchannels where they were simultaneously compressed using pneumatically actuated deformable sidewalls (Fig. 3b). Modulating the pressure in the pneumatic channels enabled precise lateral compression ranging from 0 to 22% reduction in the original embryo width. Higher levels of compression were lethal to the *Drosophila* embryos but may be desirable for other model organisms. By reversing the flow direction through the microfluidic channels, all the embryos were recovered from the system for post-stimulation analysis. Using this microsystem, we demonstrated the effect of different levels of acute and chronic compression on the embryos' developmental progression and viability. Furthermore, by fabricating this microfluidic system on a glass coverslip we were able to utilize confocal microscopy with up to a 63× objective lens to quantitatively characterize dose- and time-dependent induction of the ectopic expression of the transcription factor twist in response to mechanical compression. This design and application typify the capabilities of microfluidic systems to produce precise mechanical stimuli for mechanobiological studies at previous unattainable throughputs.

## Genetic perturbation

Ascribing a function to a gene is often complicated due to differences in the level of gene expression, alternative splicing, or localization of expression that can result in distinct phenotypic consequences. To experimentally investigate a gene's function, biologists often rely on molecular and fluorescent approaches to modulate or quantify gene expression. However, many conventional methods for genetic analysis, such as FISH and RNAi, are technically complicated, require repeated handling or injection of samples, and are low throughput, making these approaches challenging for analyzing genes with subtle phenotypic outcomes^58^. To address these limitations, Charles et al. developed a microfluidic system capable of trapping hundreds of *C. elegans* embryos and quickly enabling efficient reagent exchange (Fig. 3c)^59^. The ability to readily exchange reagents in this system allowed the researchers to perform numerous steps associated with smFISH, such as washing, hybridization, staining, and fixation, in a highly efficient manner. The small size and optically transparent materials utilized in this system also allowed for imaging with most conventional microscopy setups. While many of this device's individual features are not wholly novel, the integration of multiple features such as automated sorting, reagent exchange, and high sample throughput represents an important direction in this evolving field of microfluidics applied to biological organisms^60^. Moreover, this design typifies another trend as it can be readily scaled to accommodate samples of similar sizes such as other model organisms or organoids. Microfluidic systems can reduce the manual requirements for genetic analysis through versatility in both function and form while enabling high throughput that is statistically needed for analyzing subtle genetic phenotypes.

## Chemical stimulation

The ability to precisely control flow and generate gradients using microfluidic systems has resulted in the increased use of these tools to investigate the influence of chemical factors on biological systems^20,61^. Recent examples utilize microfluidics to study complex biological processes such as olfaction, response to toxins, and the influence of hormones, among others^62–67^. From an olfaction perspective, understanding the biological mechanisms underpinning the sense of smell is challenging due to numerous types of olfactory receptor neurons (ORNs) which, when integrated, allow animals to decode the identity and intensity of a particular odorant. Traditional methodologies to investigate olfaction are often limited to exposing a limited number of odorants often with fixed concentrations and analyzing behavioral responses^68^. To better understand how biological systems disentangle complex odorant information, Si et al. developed a microfluidic device to visualize the response of ORNs of *Drosophila* larva in response to different odorants at varied concentrations (Fig. 3d-i)^69^. Using this microfluidic system combined with three-dimensional, multi-neuronal imaging, the team characterized the responses of ORNs at the individual and population levels. This characterization determined that individual ORNs often respond to multiple odorants with different sensitivities and revealed that an odorant’s concentration would activate the same relative number of ORNs with a power-law distribution. Continued analysis using these approaches is underway to describe how downstream neurons respond to ORN excitation.

Another study utilizing *Drosophila* larvae performed by Zabihihesari et al. highlights microfluidic systems' versatility when coupled with actuators for investigating chemical stimulation^70,71^. The group developed a novel microfluidic device with integrated glass capillaries and a microneedle (Fig. 3d-ii). Given that the cardiac system is sensitive to minor environmental changes such as body orientation or time from the last feeding, studying this system requires a carefully controlled environment and precise delivery of chemical agents. Conventional methods often grapple with this inherent sensitively by dissecting out sections of the system or by multi-system techniques to investigate endogenous heart function^72,73^. In contrast, Zabihihesari et al. demonstrated that their device can gently load, orient, and immobilize larvae through the control of these integrated capillaries. Once correctly positioned in the device, larvae were injected with chemicals through a precisely located microneedle and a custom delivery system designed to limit the adverse effects of microinjection, which is often a drawback with manual injection. With this device, the group investigated hemolymph flow and serotonin's influence on the heart rate of larvae in a dose-dependent manner. This device, and similar approaches, will enable further biophysical characterization of the *Drosophila* circulatory system in addition to pharmacological and toxicological studies.

## Thermal perturbation

Temperature influences both the success and timing of development across all species. Conventional experiments have carefully characterized the development of model organisms in different temperatures, but these are typically chronic or uniform exposures to a singular temperature^74,75^. Microfluidics systems with precise spatiotemporal temperature control have enabled valuable insights into the biological mechanisms that perceive and respond to temperature changes^76^. For example, Bai et al. utilized a microfluidic device to expose a *Drosophila* embryo to a thermal gradient along the anterior-posterior axis of the embryo using two laminar flow streams with different temperatures^77^. The thermal gradient resulted in the asynchronous nuclear division along the embryo’s axis (Fig. 3e). Curiously, the team noted that the two poles show differing degrees of sensitivity to temperature cues by a yet unknown mechanism. A similar experimental paradigm was recently reported in *C. elegans* embryos that took inspiration from these *Drosophila* experiments and exposed embryos similarly to a temperature gradient with a microfluidic device^78^. In addition to observing asynchronous division across the *C. elegans* embryo, the authors also noted that the cell divisions on the warmer axis were slower than the division rate expected of an embryo exposed to media at that warm temperature uniformly. These studies highlight the capabilities of microfluidics to control flow regimes to reveal novel biological insights and highlight the ability of microfluidics to enable similar experimental paradigms across model organisms. This versatility promotes comparative analysis across phyla to better understand the functionality of conserved genes and traits.

## Design considerations and operational tips

The design and operation of microfluidic systems are extremely important as these will determine the unique functionality of the system and its ease of use, enabling new experimental paradigms to be implemented with model organisms. The systems discussed in this perspective are all designed under the physical constraints imposed at the micron scale, the material and fabrication options available at the time, and the requirement to be compatible with live or fixed biological samples. Specific experimental objectives impose additional design constraints such as a necessity to exchange reagents within the device, integration with other technologies, or material properties such as refractive index or rigidity. One challenge faced by many research groups is the need to load large numbers of individual organisms into the microfluidic systems. Poor design or operation can cause system failure due to samples adhering to the device or aggregation, resulting in a clog. Consideration of surface properties is critical to address these challenges. For example, systems have been designed with surface modification approaches to solve these problems^79^. Additional solutions include the use of mild detergent solutions or carrier fluids, such as alcohols or oils, to position the sample, followed by subsequent wash steps^80^. The use of these approaches can help to minimize the user intervention during the operation and ease the loading of samples into the microfluidic channels. Most of the microfluidic systems rely on bulky laboratory equipment (i.e., fluid pumps, bubble traps, actuators, pressure generators, and controllers) for their operation. Similarly, integration of these external tools into the microfluidic systems can make their operation more seamless, while increasing their portability^81–83^.

Another challenge faced by many designers, particularly those that incorporate mechanically active structures, is optimizing the rigidity of the materials. Overly rigid structures can reduce the precision of an immobilization system and lead to issues such as tissue damage, poor sample viability, or difficult integration with valves and actuators to control flow^14^. For many commonly used materials like PDMS, there are established fabrication and post-processing techniques that can modulate the rigidity of the resultant material, such as altering the curing duration, temperature, or curing agent ratios^84,85^. This enables the mechanically active microfluidic systems to be tailored for the given experimental goals without altering the initial design. Furthermore, these well-established and reproducible techniques can be used to facilitate the assembly of multilayered microfluidic systems with complex design^22^. Prior to utilizing a newly fabricated microfluidic system, the material properties can be tested with common instruments such as an atomic force microscope or, in many cases, the system can be functionally tested. For example, the stiffness of a PDMS layer in an immobilization chamber can be readily calculated by observing the degree of deformation at varying pressures within the chamber. Computational analysis approaches, such as finite element modeling, can help tremendously in designing microfluidic systems as these tools can identify potential points of system failure and optimize operational parameters (i.e., flow rate and profile, actuation pressure, temperature distribution, and chemical concentrations) before fabrication and experimental testing^57,86^.

The selection of material and associated fabrication techniques is critical from a design and accessibility standpoint. While designing a microfluidic system for a given experimental goal, the fabrication technique that needs to be used should be taken into account. For example, circular microchannels can easily be fabricated with micromilling while high-resolution microchannels (i.e., with future sizes less than 10 µm) typically require the use of photolithography. On the other hand, 3D printing might be a better option for multilayered microchannels or microchannels with changing height. Developments in the field of 3D printing are improving achievable print resolution and reproducibility. These continued advancements will make 3D printing a more streamlined solution for fabrication with the potential to standardize systems across labs. The most common material utilized in contemporary microfluidics is PDMS. This material has numerous advantages, such as oxygen permeability, transparency, relatively low cost, and established high-resolution fabrication techniques. Despite these advantages, there are some drawbacks to working with PDMS that make it poorly suited for some applications^87^. For example, PDMS is permeable to many solvents and organic molecules can be absorbed into the surface. This can result in autofluorescence that is incompatible with some microscopy approaches. Moreover, this can dramatically alter the concentration of chemical substances in the microfluidic channels especially if the area-to-volume ratio is high, resulting in discrepancies in chemical stimulation and cell signaling studies^88^. In cases where these concerns are prominent, a variety of different materials such as glass, poly (methyl methacrylate), polystyrene, hydrogels, thermoset polyester, and photosensitive resins are available for microfluidic fabrication. They can be used after adjusting the design of the system based on the fabrication techniques associated with these materials such as stereolithography, photolithography, micromilling, or hot embossing^89,90^. As highlighted by many examples in this perspective, we anticipate that the field of microfluidics for model organisms will expand to include a more diverse range of materials and fabrication approaches discussed above optimized for the particular requirements of a given research study.

## Outlook and future perspectives

Microfluidic technologies have enabled breakthroughs in biological research using standard biological model organisms. Among a diversity of advantages over conventional experimental approaches, such systems can enable higher throughput, precisely stimulate samples, increase reproducibility across labs, make phenotype classification more objective, eliminate the requirement for highly technical manual handling, and increase researcher efficiency through automation of mundane tasks, such as maintenance and sorting of organisms. Numerous examples of microfluidics systems designed for one particular model organism have inspired similar strategies to be developed for other model organisms. The ability to readily adapt and scale such systems to fit the requirements of one’s particular organism holds great potential for future studies and will broaden the utility of microfluidics systems across research communities. This adaptability will accelerate comparative biology analysis and empower researchers to select the best possible model organism for a particular biological phenomenon, even if it is a less-well-studied organism^12^.

The microfluidics systems described herein provide significant advantages when compared to conventional experimental approaches or create entirely new experimental paradigms. Without the aid of microfluidic systems, organisms are handled manually with tools such as tweezers, pipettes, and brushes, which is stressful to the organisms and is difficult for practitioners to master due to the small size of the organisms^91,92^. Working effectively with small samples requires hours of practice to learn how to orient samples and avoid physiological damage with gentle samples. This work is time consuming, limits throughput of experiments, increases variability, and creates a significant experience barrier to work with model organisms. These challenges are important to consider when working with live samples. Issues such as developmental progression, dehydration, physiological integrity, and intrinsic motility (with zebrafish or *Drosophila* larvae) are a few critical considerations. After samples are manually handled and oriented, they are often immobilized with adhesives or embedded in agar, which is technically challenging and can be damaging, especially to live samples^93,94^. As such, anesthetics or chemical fixation are commonly utilized techniques that, while useful, limit the experimental design^95,96^. Manually sorting model organisms typically requires a trained eye for the desired phenotype and additional handling with tools such as tweezers or pasteur pipettes. Generally, conventional techniques for handling model organisms are tedious, time-consuming, and introduce undesired mechanical stress associated with manual handling.

Likewise, stimulating samples using conventional approaches can be laborious and experimentally limiting. Many conventional approaches to stimulation necessitate that the stimulation be uniform. For example, a singular condition is conventionally applied to the entire sample or population when stimulating a sample with a given chemical agent, temperature, or altered oxygen condition^97^. In contrast, the low Reynolds number associated with the scale of microfluidics systems empowers researchers with the ability to precisely stimulate small regions or generate a gradient across a single organism^98^. Deforming organisms with bristles or micropipettes are common methods to apply mechanical stimulation without the aid of microfluidics^52,53^. Like many conventional approaches to stimulation, these methods of mechanical stimulation are typically done to a single sample at a time and are not easily accomplished with simultaneous imaging^99^. Manual injection is another common approach for fields such as toxicology and genetics. Injections are often accomplished manually, which requires a trained and steady hand and suffers from variability in injection when comparing multiple samples^100^. When compared to the conventional approaches to handling and stimulation described here, microfluidic systems provide numerous experimental advantages^101^. However, to date relatively few systems have been adopted outside of the original lab where they were developed. If the potential of these technologies is to be fully realized, the field needs to eliminate barriers to entry for model organism researchers.

A critical barrier to adopting these microfluidics solutions is the expertise associated with the manufacturing of such systems. Continued breakthroughs in materials science and fabrication techniques are making all microtechnologies significantly more accessible, including microfluidic systems^102–104^. Achieving accessibility will be accelerated by designing future microfluidics tools with an intuitive user experience in mind, minimizing the requirement for manual manipulation, automating manipulation and data acquisition, and taking advantage of automated fabrication techniques. Looking forward, automated 3D printing, holds great promise. 3D printing reduces the necessity for ancillary equipment and the user expertise often required to fabricate and operate a microsystem—a significant challenge for the adoption of microfluidic technologies^11^. Reducing or removing the requirement for on-site fabrication and designing microfluidic systems that can be readily integrated into model organisms labs is a great solution to this accessibility issue. For example, the sorting system described above by Utharala et al. details how to integrate their system with existing microscopes available in most biology labs^29^. Recent efforts to commercialize some microtechnological systems or outsource fabrication suggest that accessibility may be further increased through the commercial sector's influence as other fields utilizing microfluidics have experienced^105^. Many of the designs discussed in this perspective are open source or available upon request. Labs lacking engineering expertise to design or fabricate a novel system can readily acquire their own microfluidics system by combining these available designs with automated or outsourced fabrication solutions such as 3D printing or commercially available microfluidic manufacturers. Selecting materials and fabrication techniques that non-experts can implement and designing for intuitive user experience is critical for the adoption of microfluidic technologies by researchers, including biologists in model organism communities. These convergent trends are quickly making microfluidics systems more accessible and, in doing so, empowering researchers with a tremendous number of benefits, as highlighted in this perspective.

Microfluidic systems' effectiveness in biological research will be further increased through their unique combination with other state-of-the-art technologies. Early examples of such hybrid systems include coupling microfluidics with machine learning, light-sheet microscopy, robotics, and genomics technologies, and provide a glimpse at the potential to facilitate the examination and analysis of spatiotemporal dynamics of cells within intact organisms^103,106^. For example, Mattern et al. recently developed an all-glass microfluidic device, dubbed NeuroExaminer, that enabled live imaging of whole zebrafish larval brains at single-cell resolution using light-sheet microscopy^107^. We anticipate that similar microfluidics systems will be used in future work to acquire volumetric imaging data quickly without sacrificing or harming organisms^108^. Such an approach would permit repeated imaging of an individual organism to investigate dynamic neurological or developmental processes over time. Another example of combining microfluidics with other state-of-the-art technologies can be seen in Gizzi et al.^109^ Their team developed a microfluidics system coupled with a robotic microscope setup for sequential imaging and oligopaint technologies to simultaneously observe chromatin organization and quantify transcription within a single nucleus of an intact, whole *Drosophila* embryo. Finally, Atakan et al. developed a microfluidics system capable of sorting hundreds of *C. elegans* embryos, precisely exposing the samples to stimuli via controlled flow through the system, and automatically phenotyping the embryos throughout development by coupling image processing and deep learning approaches^110^. These examples suggest a promising trajectory for the field, one in which microfluidic systems are designed for automation, high-throughput, and precise stimulation to produce massive data sets ideal for statistical power, objective interpretation through the use of data analysis algorithms, and novel biological insights from machine learning.

## Conclusions

These tools open doors for more quantitative studies of model organisms, continuing to provide insights into developmental biology, ethology, neurobiology, biophysics, and biologically inspired engineering. As these tools become more accessible and further integrated with other state-of-art technologies, model organism communities will benefit from the advantages of microfluidics and promote the creation of novel experimental paradigms.

Model organism research utilizing microfluidic technologies has produced novel biological insights and tremendous advances in experimental approaches, including increased throughput, automated sample manipulation, precise stimulation, improved reproducibility, and improved portability. Continued transdisciplinary collaboration between biologists and engineers is accelerating the rate of adoption of these tools by the model organism research communities and boosting the realization of the great potential of microfluidic systems applied to model organism research. Microfluidic tools are becoming more accessible through improvements in fabrication, ease of use, and the ability to readily scale and tailor the design from one model organism to another. Integration of microfluidics with additional state-of-the-art technologies such as robotics, machine learning, and light-sheet microscopy will further expand the experimental capabilities and utility of model organisms. Microfluidic tools augment the potential of model organism research and, in doing so, accelerate our understanding of the mechanisms underpinning life, which are applicable in a diversity of fields in the future, from human disease to understanding evolution to future space colonization.
